# Assessment of the requisites of microbiology based infectious disease training under the pressure of consultation needs

**DOI:** 10.1186/1476-0711-10-38

**Published:** 2011-12-16

**Authors:** Hakan Erdem, Suda Tekin-Koruk, Ibrahim Koruk, Derya Tozlu-Keten, Aysegul Ulu-Kılıc, Oral Oncul, Rahmet Guner, Serhat Birengel, Gurkan Mert, Saygin Nayman-Alpat, Necla Eren-Tulek, Tuna Demirdal, Nazif Elaldi, Cigdem Ataman-Hatipoglu, Emel Yilmaz, Bilgul Mete, Behice Kurtaran, Nurgul Ceran, Oguz Karabay, Dilara Inan, Melahat Cengiz, Suzan Sacar, Behiye Yucesoy-Dede, Sibel Yilmaz, Canan Agalar, Yasar Bayindir, Yesim Alpay, Selma Tosun, Hava Yilmaz, Hurrem Bodur, Huseyin A Erdem, Nebahat Dikici, Murat Dizbay, Serkan Oncu, Nurbanu Sezak, Tuba Sari, Oguz R Sipahi, Serhat Uysal, Esma Yeniiz, Selcuk Kaya, Asim Ulcay, Halil Kurt, Bulent A Besirbellioglu, Haluk Vahaboglu, Yesim Tasova, Gaye Usluer, Dilek Arman, Husrev Diktas, Sercan Ulusoy, Hakan Leblebicioglu

**Affiliations:** 1 Kasımpasa Hospital, Department of Infectious Diseases and Clinical Microbiology (IDCM), Istanbul, Turkey; 2 Harran University, School of Medicine, Department of IDCM, Sanliurfa, Turkey; 3 Harran University, School of Medicine, Department of Public Health, Sanliurfa, Turkey; 4 Gazi University, School of Medicine, Department of IDCM, Ankara, Turkey; 5 Erciyes University, School of Medicine, Department of IDCM, Ankara, Turkey; 6 Gulhane Haydarpasa Hospital, Department of IDCM, Istanbul, Turkey; 7 Ataturk Training and Research Hospital, Department of IDCM, Ankara, Turkey; 8 Ankara University, School of Medicine, Department of IDCM, Ankara, Turkey; 9 Gulhane Medical Academy, Department of IDCM, Ankara, Turkey; 10 Osmangazi School of Medicine, Department of IDCM, Eskisehir, Turkey; 11 Ankara Training and Research Hospital, Department of IDCM, Ankara, Turkey; 12 Kocatepe School of Medicine, Department of IDCM, Afyon, Turkey; 13 Cumhuriyet School of Medicine, Department of IDCM, Sivas, Turkey; 14 Uludag School of Medicine, Department of IDCM, Bursa, Turkey; 15 Cerrahpasa School of Medicine, Department of IDCM, Istanbul, Turkey; 16 Cukurova School of Medicine, Department of IDCM, Adana, Turkey; 17 Haydarpasa Numune Training and Research Hospital, Istanbul, Turkey; 18 Sakarya School of Medicine, Department of IDCM, Sakarya, Turkey; 19 Akdeniz School of Medicine, Department of IDCM, Antalya, Turkey; 20 Maltepe School of Medicine, Department of IDCM, Istanbul, Turkey; 21 Pamukkale School of Medicine, Department of IDCM, Denizli, Turkey; 22 Uskudar State Hospital, Department of IDCM, İstanbul, Turkey; 23 Ataturk School of Medicine, Department of IDCM, Erzurum, Turkey; 24 Kirikkale School of Medicine, Department of IDCM, Kırıkkale, Turkey; 25 Inonu School of Medicine, Department of IDCM, Malatya, Turkey; 26 Cengiz Gokcek State Hospital, Department of IDCM, Gaziantep, Turkey; 27 Manisa State Hospital, Department of IDCM, Manisa, Turkey; 28 Ondokuzmayıs School of Medicine, Department of IDCM, Samsun, Turkey; 29 Numune Training and Research Hospital, Department of IDCM, Ankara, Turkey; 30 Ege School of Medicine, Department of IDCM, İzmir, Turkey; 31 Selcuklu School of Medicine, Department of IDCM, Konya, Turkey; 32 Adnan Menderes School of Medicine, Department of IDCM, Aydin, Turkey; 33 Girne Military Hospital, Department of IDCM, Girne, Turkey; 34 Karadeniz School of Medicine, Department of IDCM, Trabzon, Turkey; 35 Kocaeli School of Medicine, Department of IDCM, Kocaeli, Turkey

**Keywords:** Infectious disease, clinical microbiology, training, consultation

## Abstract

**Background:**

Training of infectious disease (ID) specialists is structured on classical clinical microbiology training in Turkey and ID specialists work as clinical microbiologists at the same time. Hence, this study aimed to determine the clinical skills and knowledge required by clinical microbiologists.

**Methods:**

A cross-sectional study was carried out between June 1, 2010 and September 15, 2010 in 32 ID departments in Turkey. Only patients hospitalized and followed up in the ID departments between January-June 2010 who required consultation with other disciplines were included.

**Results:**

A total of 605 patients undergoing 1343 consultations were included, with pulmonology, neurology, cardiology, gastroenterology, nephrology, dermatology, haematology, and endocrinology being the most frequent consultation specialties. The consultation patterns were quite similar and were not affected by either the nature of infections or the critical clinical status of ID patients.

**Conclusions:**

The results of our study show that certain internal medicine subdisciplines such as pulmonology, neurology and dermatology appear to be the principal clinical requisites in the training of ID specialists, rather than internal medicine as a whole.

## Introduction

Infectious diseases (ID) specialists either directly manage infections or they provide consultation service to other hospital departments. Patients with infections, seen by ID consultants, are more likely to receive effective and appropriate empirical antimicrobial therapy, to have decreased treatment costs, to survive the infection and be cured [1-4]. Referring clinicians expect ID specialists to mainly focus on recommendations for prompt and accurate methods to diagnose infection, design local hospital antibiotic policies to facilitate appropriate antimicrobial therapy and prophylaxis, surveillance of antimicrobial resistance and hospital epidemiology, and the establishment of hospital infection control programs [5]. On the other hand, as physicians working in their own departments, ID specialists are required to meet all the clinical needs of ID patients, not only the provision of infection management.

In medical practice much of the work done by both ID physicians and clinical microbiologists requires both laboratory and clinical skills. Supporters of combined ID and clinical microbiology practice are already known in the world. For instance, according to Peter Moss, the vice-president of the British Infection Association, there are proposals to bring the two training curricula together in the United Kingdom in the future. In Turkey, ID physicians come from a laboratory background and have been working as ID and Clinical Microbiology (IDCM) specialists (IDCMSs). According to regulations implemented in 2010 by Turkish Medical Postgraduate Training Council, a total of five-year ID training in Turkey involved 12 months of internal medicine, two months of pulmonology, and one month of pediatrics teaching [6]. Before that date internal medicine made up a total of six months in the postgraduate ID training calendar in Turkey. However, this was not applied compulsorily in most of the training hospitals, but rather accepted as an advice before 2010 in Turkey. On the other hand, every ID department has its own laboratory inside the clinic, and the IDCM fellows have been trained in the field of microbiology in due course of all their training at the bench side. Thus, this study aims to provide data to determine which medical disciplines are frequently necessary to fulfil the needs of patients hospitalized in ID clinics, and at which point the IDCMSs need to consult with other discipline specialists. Consequently, our study aims to identify possible ways to strengthen ID training, which is and should be interrelated to microbiology. The idea behind this was that how an IDCM fellow could be trained in other medical fields to offer perfect medical service in the treatment of an ID patient with coexistent disorders or for those the infections caused considerable organ based injuries. That point of view did not target the utopia for IDCMSs to substitute clinicians from other medical disciplines. Rather, we believe that this approach will help the optimization of the processes like referring the patients to other specialists, the decision of optimal timing of consultation, compliance to their recommendations, and the adaptability of the IDCMSs into the changing status of the patient either due to concordant non-ID problems or owing to the ID-based organ injuries. In addition, combining the curricula of other medical branches into the ID training will contribute handling the situation by IDCMSs' own to a degree at the minimum. We believe that this evidence-based training policy will decrease the workload in the hospital and purify the interdisciplinary collaborations. On the other hand, to the best of the authors' knowledge, this is the first study of its kind in the literature on the utilization of specialty consultation services provided to ID departments.

### Patients and methods

This cross-sectional study was carried out between June 1, 2010 and September 15, 2010 in 32 ID departments in Turkey. Four of these clinics were in public training hospitals, four of them were in public hospitals, and 24 were in university hospitals. Patients who were hospitalized in ID departments and required consultation with specialists of other disciplines between January-June 2010 were included in the study. ID patients who did not receive consultation with specialists of other disciplines were excluded.

A questionnaire, which evaluated the consultation process and included an excel file for collecting institutional data, were delivered to participant centres. Patients with fever of unknown origin were excluded from the study since these cases are routinely seen in various disciplines. Primary diagnosis was defined as the dominant clinical presentation and secondary diagnosis was any relatively less important clinical condition according to the evaluation of the IDCMSs who admitted the patient to the hospital.

The IDCMSs were never in charge of intensive care unit (ICU) in Turkey, but rather they provide consultation service to these departments. Sepsis, severe sepsis, septic shock, invasive or noninvasive mechanical ventilation, and ICU admission were the parameters used for the evaluation of the critical status [7].

The patients with infection related final diagnoses (IRFD) confirmed at the end of the consultation process were given special consideration with the understanding that this subgroup of patients would be treated primarily by the IDCMSs. In patients with IRFD, the altered initial diagnoses of IDCMSs and the establishment of the diagnoses at the end of consultation process were accepted as a sole group in which the consultants contributed to final diagnoses (D1). Similarly, unchanged initial diagnoses of IDCMSs where the consultants did not contribute to diagnosis were accepted as the other group (D2). According to therapeutic approaches for patients with IRFD, starting the treatment, changing the regimen or addition of drugs to the initial regimen at the end of the consultation process were accepted as the sole group where the consultant contributed to treatment (T1). Continuation of the initial treatment without modification where the consultant did not contribute to therapy was recognized as the other group for patients with IRFD (T2).

In this study, health care-associated infection (HCAI), also referred to as "nosocomial" or "hospital" infection, was defined as an infection occurring in a patient during the process of care in the hospital. By definition, this infection was not present or incubating at the time of hospital admission [8]. On the other hand, community-acquired infection was defined as a disease, which took place in daily life in the community and by definition; it should be unrelated to HCAI.

Statistical analyses were performed by SPSS 11.5 software program. Mann-Whitney (M-W) U, Kruskall Wallis, Mantel Haenszel, Chi Square, Kendall's tau, Pearson correlation tests and descriptive statistics were used. P-values lower than 0.05 was accepted as statistically significant. The dependent variable in our study was the number of consultations. The median consultation numbers in severe sepsis, septic shock and ICU patients, as the critical cases, were two. For this reason, this point was accepted as the cut-offs as less than and equal to two (infrequent consultation requests), and more than and equal to three [frequent consultation requests (FCR)]. Thus, dependent variables were turned into categorical data and bivariate analyses were performed. A logistic regression model was established to evaluate the real significance of significant variables detected in the bivariate analyses.

## Results

In this study, 1343 consultations belonging to 605 patients were included. IDCMSs requested 815 consultations (60.7%) from medical disciplines and 528 consultations (39.3%) from surgical clinics. Seventy-nine patients were hospitalized for two distinct infections and 526 cases had just one ID diagnosis. When patients were evaluated according to sepsis definitions, 148 (24.5%) were defined as having sepsis, 130 (21.5%) had severe sepsis, and 18 (3.0%) had septic shock. The evaluation on patients' outcomes indicated that, 363 cases (60.0%) were discharged with complete cure, 131 (21.7%) were sent home after clinical improvement or with sequential therapy, 59 cases (9.8%) were transferred to another department in the same hospital, 26 patients (4.3%) died, 16 cases (2.6%) were transferred to another hospital, and ten cases (1.6%) took their own discharge. Among the patients transferred to another department, 20 (3.3%) cases were assigned to medical disciplines and 39 (6.5%) were transferred to surgical departments. General surgery was the most frequent transfer location area with 12 cases, followed by five patients transferred to neurosurgery. When the consultation requests were assessed owing to invasive expectations of the IDCMSs or due to either symptom or syndrome-based grounds, IDCMSs consulted surgical departments for invasive procedures more frequently than medical departments (χ^2 ^= 12.340, P **= 0.002**).

The consultant departments are presented in Table 1 and the distribution of referred clinics according to ID diagnoses for which the patients were hospitalized are presented in Table 2. Analyses for FCR are shown in table 3 and the independent variables affecting FCR are assessed in a logistic regression model, which is presented in table 4.

**Table 1:** Distribution of consultations for patients hospitalized in infectious diseases departments by type of patient, department and final diagnose

CONSULTANT CLINICS	Sepsis Patients	ICU Patients	Final Diagnoses of the Consultations					Comrb
					
			Related to ID		Unrelated to ID		Total	
					
			n	%	n	%		
Pulmonology	53	6	61	(57.5%)	45	(42.5%)	106	15

Neurology	51	7	49	(51.0%)	47	(49.0%)	96	13

General Int Med	45	4	46	(45.4%)	49	(51.6%)	95	

Cardiology	40	9	59	(67.8%)	28	(32.2%)	87	117

Gastroenterology	24	5	36	(59.0%)	25	(41.0%)	61	17

Nephrology	22	2	32	(52.5%)	29	(47.5%)	61	28

Dermatology	10	9	31	(55.4%)	25	(44.6%)	56	

Haematology	22	3	30	(68.2%)	14	(31.8%)	44	13

Endocrinology	22	3	29	(65.9%)	15	(34.1%)	44	49

Psychiatry	20	5	31	(73.8%)	11	(26.2%)	42	1

Physical Ther & Rehab	10	3	19	(54.3%)	16	(45.7%)	35	6

Oncology	12	1	3	(21.4%)	11	(78.6%)	14	20

Rheumatology	7	3	8	(57.1%)	6	(42.7%)	14	

Immunology	3		6	(66.7%)	3	(33.3%)	9	1

**Medical Clinics Subtotal**			**440**	(57.6%)	**324**	(42.4%)	**764**	

ENT Department	36	3	35	(53.0%)	31	(47.0%)	66	

General Surgery	34	4	46	(57.5%)	34	(42.5%)	80	

Urology	33	9	36	(51.4%)	34	(48.8%)	70	28

Orthopaedics	23	6	46	(66.6%)	23	(33.3%)	69	

Ophthalmology	23	3	32	(60.4%)	21	(39.6%)	53	

Plastic Surgery	20	4	20	(60.6%)	13	(39.4%)	33	

Neurosurgery	18	4	36	(69.2%)	16	(30.8%)	52	14

Cardiovascular Surgery	17	3	18	(52.9%)	16	(47.1%)	34	9

Anaesthesiology	17	4	30	(65.2%)	16	(34.8%)	46	

Gynaecology	13	1	15	(57.7%)	11	(42.3%)	26	

Thoracic Surgery	8	1	8	(57.1%)	6	(42.9%)	14	

Dentistry	7		9	(69.2%)	4	(30.8%)	13	

Hyperbaric Oxygen Unit	2		1	(50.0%)	1	(50.0%)	2	

Radiology	10		9	(50.0%)	9	(50.0%)	18	

Diet Department	3		1	(33.3%)	2	(66.6%)	3	

**Surgical Clinics Subtotal**			342	(59.0%)	237	(41.0%)	579	

**TOTAL**	**296**	**41**	**782**	(58.3%)	**561**	(41.8%)	**1343**	

**ICU**: Intensive Care Unit **Int Med: **Internal Medicine **ID:**Infectious diseases, **Non-ID:**Noninfectious disease, **ENT: **Ear-Nose-Throat, **Physical Ther & Rehab: **Physical Therapy &Rehabilitation, **Comrb: **Comorbid conditions other than infectious diseases. Data are classified according to related departments.

**Table 2:** Percentages of the most frequent consulting clinics according to infection diagnoses

Lower Respiratory Tract Infections (n:118)	Pulm-tbc (n:48/6)*	Gstr (14.6)	GenS (12.2)	Pulm (9.8)	Neph (9.8)	Gn-IM (7.3)	Hema (6.1)	Card (4.9)	Neur (4.9)	TxS (3.7)	Orth (3.7)
	
	Pneumonia (n:64)	Pulm (23.9)	Card (9.4)	Gn-IM (5.8)	Gstr (5.8)	Neur (5.8)	ENT (5.8)	GenS (5.8)	Uro (4.3)	Anes (3.6)	Endc (3.6)
**Upper respiratory infections** **(n:9)**		Pulm (26.7)	Hema (20.0)	ENT (13.3)	Endc (6.7)	Gn-IM (6.7)	Neph (6.7)	Onco (6.7)	Neur (6.7)	PhTR (6.7)	

**Herpetoviridae infections** (n:30/28)*		Pulm (14.0)	Derm (12.0)	Gn-IM (10.0)	Card (10.0)	Rad (6.0)	Opht (6.0)	GenS (4.0)	Uro (4.0)	ENT (4.0)	Neur (4.0)

**Central Nervous System Infections** **(n:75)**	**Tbc- Men** (n:11/3)*	Neur (27.8)	NeuS (15.8)	Opht (13.9)	Pulm (8.3)	TxS (5.6)	Hema (5.6)	Gn-IM (2.8)	Psyc (2.8)	ENT (2.8)	Neph (2.8)
	
	**Acute-Men** **(n:36)**	Neur (18.3)	NeuS (15.6)	ENT (11.9)	Gn-IM (10.1)	Anes (7.3)	Pulm (7.3)	Opht (6.4)	Card (4.6)	Psyc (2.8)	Endc (1.8)
	
	**Encephalitis** (n:11/14)*	Neur (48.3)	NeuS (6.9)	Opht (6.9)	GenS (6.9)	Gn-IM (3.4)	ENT (3.4)	Psyc (3.4)	Card (3.4)	Rhm (3.4)	Endc (3.4)

Extra-pulmonary tuberculosis (n:8/1)*		NeuS (15.8)	GenS (10.5)	Derm (10.5)	Card (10.5)	Neur (5.3)	ENT (5.3)	Anes (5.3)	Orth (5.3)	Pulm (5.3)	TxS (5.3)

**Viral Hepatitis** **(n:34)**	Acute (n:18/1)*	Gstr (50.0)	Hema (15.0)	Gn-IM (10.0)	GenS (10.0)	Pulm (5.0)	Gyn (5.0)	PhTR (5.0)			
	
	**Chronic** (n:9/6)*	Gstr (28.6)	Gn-IM (14.3)	Pulm (14.3)	ENT (14.3)	Opht (7.1)	Hema (7.1)	Psyc (7.1)	Endc (7.1)		
	
**Urinary infections** **(n:110)**		Uro (21.2)	Neph (10.2)	Gn-IM (9.3)	Neur (6.7)	GenS (6.6)	Gyn (5.8)	Card (5.8)	Pulm (4.9)	Endc (4.4)	Anes (3.1)

**Bone-joint infections** **(n:50)**		Orth (19.8)	Anes (9.0)	PlaS (7.7)	Derm (7.2)	NeuS (6.6)	Psyc (6.6)	PhTR (5.4)	Endc (5.4)	CVS (3.6)	Neph (3.6)

**Skin and soft tissue infections** **(n:94)**		Orth (7.8)	GenS (7.8)	ENT (7.8)	Derm (7.3)	PlaS (6.8)	Pulm (6.8)	Card (6.8)	CVS (6.8)	Gn-IM (6.4)	PhTR (3.7)

**Gastrointestinal infections** **(n:10)**		GenS (35.0)	Gstr (25.0)	Gn-IM (20.0)	Derm (10.0)	Psyc (10.0)					

**Abscesses** (n:12/1)*		GenS (16.7)	Rad (12.5)	Pulm (12.5)	NeuS (8.3)	Onco (8.3)	Neur (8.3)	Endc (4.2)	Gstr (4.2)	ENT (4.2)	Card (4.2)

**Endocardial infections** **(n:15)**		Card (23.5)	CVS (13.7)	Neur (9.8)	Gn-IM (5.9)	Opht (5.9)	Pulm (5.9)	Derm (5.9)	Dent (3.9)	Neph (3.9)	ENT (3.9)

Congo-Crimean haemorrhagic fever (n:22/2)*		Hema (20.0)	Gn-IM (13.3)	ENT (13.3)	GenS (10.0)	Endc (10.0)	Gyn (6.7)	Neur (6.7)	Dent (3.3)	Uro (3.3)	Gstr (3.3)

**Invasive Fungal infections** **(n:9)**		Hema (21.1)	Neph (10.5)	Card (10.5)	Gn-IM (5.3)	Imm (5.3)	Anes (5.3)	Derm (5.3)	Uro (5.3)	GenS (5.3)	Pulm (5.3)

**Zoonoses** (n:29/5)*		Gn-IM (15.2)	Card (13.0)	NeuS (8.7)	ENT (8.7)	Opht (6.5)	Psyc (6.5)	Orth (4.3)	GenS (4.3)	Neur (4.3)	PhTR (4.3)

**HIV infections** (n:12/12)*		Opht (27.6)	Derm (13.8)	Gn-IM (6.9)	Pulm (6.9)	Psyc (6.9)	Anes (6.9)	Orth (3.4)	PhTR (3.4)	GenS (3.4)	Neph (3.4)

*Primary diagnosis/Secondary diagnosis, Pulm-tbc: Pulmonary tuberculosis, Acute-Men: Acute Meningitis

Gstr: Gastroenterology, GenS: General surgery, Pulm: Pulmonology, Neph: Nephrology, Gn-IM:General-internal medicine, Hema: Haematology, Card: Cardiology, Neur: Neurology, TxS: Thoracic Surgery, Orth: Orthopaedics, NeuS: Neurosurgery, Opht: Ophthalmology, Psyc: Psychiatry, ENT: Ear-nose-throat, Derm: Dermatology, Anes: Anaesthesiology, Endc: Endocrinology, Uro: Urology, Rhm: Rheumatology, Gyn: Gynaecology, PhTR: Physical Therapy and Rehabilitation, CVS: Cardiovascular Surgery, PlaS: Plastic Surgery, Onco: Oncology, Rad: Radiology, Dent: Dentistry, Imm: Immunology

**Table 3:** Factors affecting frequent consultation requests in bivariate analyses

Variables		3 and upper		2 and lower		Significance	
		**N**	**%**	**n**	**%**	**χ^2^**	***P***

**Gender**	Female	225	39.2	349	60.8	**3.90**	**0.048**
			
	Male	260	33.8	509	66.2		

**Nature of ID**	CAI	390	34.8	732	65.2	**5.06**	**0.024**
			
	HCAI	95	43.0	126	57.0		

**Type of Hospital**	Public	15	15.0	85	85.0	**30.97**	**< 0.001**
			
	Public training *	166	32.6	343	67.4		
			
	University *	304	41.4	430	58.6		

**Source of patient**	Emergency	174	34.3	333	65.7	**18.2**	**< 0.001**
			
	ID polyclinic	184	32.7	378	67.3		
			
	Other clinic *	64	43.5	83	56.5		
			
	Outer centre *	64	50.3	63	49.7		

**Number of IDs**	One ID	374	34.0	725	66.0	**10.87**	**0.001**
			
	Two IDs	111	45.5	133	54.5		

**Number of comorbidities**	None	215	31.9	460	68.1	**20.61**	**< 0.001**
			
	One disorder	145	35.7	261	64.3		
			
	Two disorder	125	47.7	137	52.3		

Site of infection**	CNS*	87	49.7	88	50.3	**66.34**	**< 0.001**
			
	Endocard tissue*	25	49.0	26	51.0		
			
	Skin-soft tissue*	86	40.4	127	59.6		
			
	Abscesses*	11	39.3	17	60.7		
			
	EP-tbc*	7	33.3	14	66.7		
			
	UTI*	70	31.1	155	68.9		
			
	Gastrointestinal*	6	30.0	14	70.0		
			
	Other	193	31.6	417	68.4		

**Sepsis status**	None	213	33.3	426	66.7	**10.20**	**0.017**
			
	Sepsis	111	35.2	204	64.8		
			
	Severe sepsis*	150	42.9	200	57.1		
			
	Septic shock	11	28.2	28	71.8		

**ICU status**	Yes	47	41.6	66	58.4	1.35	0.24
			
	No	438	35.6	792	64.4		

**NIMV**	Yes	11	32.4	23	67.6	0.07	0.77
			
	No	474	36.2	835	63.8		

**MV**	Yes	29	46.0	34	54.0	2.38	0.12
			
	No	456	35.6	824	64.4		

**O2 by nasal mask**	Yes	127	46.9	144	53.1	**16.42**	**< 0.001**
			
	No	358	33.4	714	66.6		

**Discharge type**							

Complete cure		165	26.4	461	73.6	**12.72**	**0.026**
				
With improvement or sequential therapy		80	29.7	189	70.3		
				
Transferring to other clinic		31	27.9	80	72.1		
				
Taking own discharge		5	27.8	13	72.2		
				
Transferring to other hospital		16	41.0	23	59.0		
				
Death*		29	44.6	36	55.4		

**Age**		**n**	**Mean ± sd**	**n**	**Mean ± sd**	**M-W U**	***P***
		
		485	54.7-18.5	858	49.9-20.0	**178514**	**< 0.001**

**Length of hospital stay**		485	29.4-20.1	858	17.2-13.7	**123869**	**< 0.001**

*The group which contributes to significance, **The analyses were made one by one or all infection sites, but for convenience insignificant parameters were unified as others in the table

**Table 4:** Factors associated with frequent consultation requests in multivariate analyses

	B	*P*	OR	95% CI
Gender (Female)	**0.534**	**< 0.001**	**1.7**	**1.26-2.29**
Nature of ID (HCAI)	0.026	0.89	1.02	0.69-1.52

Type of hospital (public training)	**0.97**	**0.004**	**2.65**	**1.36-5.18**

Type of hospital (university)	0.61	0.07	1.84	0.94-3.60

Source of patient (Other clinic)	0.37	0.12	1.45	0.90-2.34

Source of patient (Outer centre)	0.26	0.32	1.29	0.77-2.17

Number of IDs (Two)	-0.14	0.51	0.86	0.56-1.32

Number of comorbidity (one disorder)	0.05	0.77	1.05	0.73-1.52

Number of comorbidity (two disorder)	**0.58**	**0.005**	**1.78**	**1.19-2.67**

Site of infection (CNS)	-0.14	0.54	0.86	0.53-1.38

Site of infection (endocard)	-0.08	0.84	0.91	0.39-2.14

Site of infection (skin-soft tissue)	0.10	0.66	1.10	0.69-1.76

Site of infection (abscesses)	-0.15	0.76	0.85	0.31-2.36

Site of infection (EP-Tbc)	-20.66	0.99	0.00	0.00-

Site of infection (UTI)	0.001	0.99	1.001	0.63-1.57

Site of infection (gastrointestinal)	0.43	0.44	1.54	0.51-4.60

Sepsis status (severe)	0.24	0.16	1.27	0.90-1.80

O2 by nasal mask (yes)	0.12	0.58	1.12	0.73-1.73

Discharge type (death)	0.67	0.059	1.95	0.97-3.93

Age	**0.01**	**0.01**	**1.01**	**1.003-1.01**

Length of hospital stay	**0.03**	**< 0.001**	**1.03**	**1.02-1.04**

**HCAI: **Health care associated infection, **UTI: **Urinary tract infection, **EP-Tbc: **Extra-pulmonary tuberculosis

The most frequently contacted medical disciplines were pulmonology, neurology, general internal medicine, cardiology, gastroenterology, nephrology, dermatology, haematology, and endocrinology while ear-nose-throat (ENT), general surgery, urology, orthopaedics, ophthalmology, and neurosurgery were the most commonly needed surgical clinics.

When D1 and D2 groups were compared for patients with IRFD, IDCMSs have significantly higher unchanged initial diagnoses in urology consultations (% 59.6) (χ^2 ^= 4.226, **P = 0.040**). Therapeutic approaches after the establishment of definite diagnosis such as starting treatment, changing the regimen, addition of drugs to the initial regimen or continuing the initial treatment without modification were not related to higher consultation demands (χ^2 ^= 7.17, P = 0.06). There was a significant difference for T1 and T2 groups between the departments (Mantel Haenszel χ^2 ^= 29.16, **P = 0.000**). IDCMSs have significantly higher unchanged therapeutic approaches for patients with IRFD in the consultations of general internal medicine (66.0%) and urology (66.7%) (χ^2 ^= 10.106, **P = 0.001; **χ^2 ^= 4.707, **P = 0.030 **respectively).

## Discussion

Infectious Diseases training is commonly accepted to be a combination of clinical microbiology, internal medicine and epidemiology [9-12]. ID clinicians possess an array of valuable skills. Experienced ID physicians often reduce the use of unnecessary expensive diagnostic tests; use the outpatient field for continued intravenous therapy; switch to sequential oral therapy when appropriate; and enhance patient satisfaction by optimizing the overall quality [9]. The question is how to provide the relevant training to achieve these skills. The optimum training design should be based on the needs of, and fit well within the overall health structure of the country. For this reason, the specialty of ID has developed differently in different countries over the years [11]. The purpose of this paper was not either to advocate any particular design or to evaluate the lacking skills in a qualitative way. But, rather to focus on the clinical requisites or the dependency of the ID specialist training based on clinical microbiology.

Patient flow in the ID department is mainly thorough the ID polyclinic, where the patients first applied in the hospital. This is followed by the other patients transferred from the other departments including the emergency room. According to Guven Celebi, who worked on a survey on the remuneration of IDCMSs in Turkey, these doctors are generally paid in 2000 to 3000 Euros range depending on the workload in Turkish Public and University Hospitals.

According to our data pulmonology support was the most frequent requisite of hospitalized ID cases followed by neurology. The cooperation requirements were also clear for other internal medicine disciplines such as cardiology, gastroenterology, nephrology, hematology, and endocrinology. Dermatology support was frequently sought and IRFD comprised more than half of the cases for that discipline. IDCMSs were found to have better therapeutic approaches for the patients with IRFD who were consulted to general internal medicine. That department is the primary application site of all internal medicine disciplines and the patients are distributed to other internal medicine clinics via general internal medicine. Hence, IDCMSs had better patient management for ID patients when they were to consult general internal medicine, probably due to the relatively basic nature of this branch. Another standpoint was that ID patients needed 1.8 fold more frequent external help when the coexisting noninfectious disorders increased from one to two. In our patients the most frequent comorbid noninfectious conditions were related to cardiology, nephrology, and endocrinology. Some concordant disorders have the particular potential ID impacts, as in diabetes mellitus [13] or chronic renal insufficiency [14]. According to our data, IDCMSs seldom needed oncology, rheumatology and immunology for their patients. Consequently, it appears that these clinics and general internal medicine are not the principal skills for IDCMSs in patient management.

In this study, ENT department, general surgery, and urology were the most frequent surgical contacts in supervising ID patients. Therapeutic and diagnostic approaches were significantly better in patients with IRFD in urology consultations probably due to the fact that urinary infections are common in hospitals [15] and that increasing awareness of IDCMSs had already been established on this issue and related subjects. In more than half of the consultations provided by all surgical departments, the final diagnoses of the patients were related to ID and the IDCMSs referral was largely for invasive procedures. This occurred less commonly in referrals to other medical disciplines. This is consistent with the historical need for surgeons in ID patient management where surgical intervention is required to control or eliminate infection. Moreover, two-thirds of the hospitalized ID patients who were transferred to another department were passed on to surgical clinics, general surgery being the most frequent one. That is, 6.5% of our ID patients were transferred to surgeons. Obviously, these frequent interrelations with surgeons cannot be inferred as combining the curricula of surgical clinics into ID training. But rather, strengthening ID training in aforementioned medical areas may contribute optimal viewpoints for their surgical counterparts as in neurology and neurosurgery, pulmonology and thoracic surgery, cardiology and cardiovascular surgery, nephrology and urology, and finally, gastroenterology and general surgery.

According to our data, when hospitalized ID patients had additional infectious diagnoses, consultation demands of IDCMSs were not enhanced. Moreover, the consultation patterns were similar for both HCAIs and community-acquired infections and they did not significantly vary between the major ID clinical syndromes. In routine ID practice, infections pose a formidable challenge particularly due to resistance issues or highly virulent microorganisms [16-18]. But the results of this study indicated that an infection of any origin in an ID department required similar external help. Accordingly, the hospital admission source of the patients or discharge types including death did not increase consultation demands in due course of hospitalization. As expected, increased length of hospital stay and advancing age slightly increased the consultation needs. Interestingly, being a female increased consultation demands according to our data. The reason for this finding is unclear and needs further clarification. Consequently, when the patient was once accepted in an ID clinic, the IDCMSs had uniform approaches in consulting to other departments. However, a major difference was seen in the institutions where the patients were hospitalized. Training hospitals are generally more well-equipped institutions and enriched with many medical sub-disciplines not possible in ordinary public hospitals in Turkey. It appears that the IDCMSs found it easier to consult with other disciplines in training hospitals, and according to our study they requested consultation services 2.7 times more frequently than in public hospitals.

In this study, we evaluated the critical status of the patients to disclose whether consultation demands were affected in this particular subgroup of cases or not. According to the results of our logistic regression model, presences of sepsis, severe sepsis, septic shock, administration of the oxygen by nasal cannula or by a mask, noninvasive mechanical ventilation, invasive mechanical ventilation, and ICU admission in an ID patient did not enforce the patient's doctor to seek additional help and the behaviour of IDCMSs were homogeneous in either critical or non-critical ID patients.

Historically, the practice of ID and clinical microbiology come from a common origin in Turkey. In 1929 this ancestral branch was referred to as "Bacteriology" in the National Medical Specialization Act followed by "Bacteriology and Infectious Diseases" according within the 1947 regulations. Finally in 1983, the discipline was defined as "Infectious Diseases and Clinical Microbiology" [19]. According to our data, some of the internal medicine disciplines, plus pulmonology, neurology and dermatology are principal clinical requisites in the training of laboratory based ID specialists, rather than internal medicine as a whole. Moreover, the results of our study showed that consultation habits of IDCMSs are quite homogenous and not affected by either the nature of infections or the status of ID patients. It appears that IDCMSs have uniform consulting behaviours in the management of critical ID patients, and the routine training programs of aforementioned disciplines seemingly may contribute to this issue. As a result, in providing better patient care, optimal follow-up, and for more professional collaboration with frequently contacted clinics, combining the curricula of these disciplines with the ID training appears to be a rational strategy.

## Competing interests

The authors declare that they have no competing interests.

## Authors' contributions

FG conceived of the study, and participated in its design and coordination. HE designed and coordinated the study. ST-K helped designing and coordinating the study. IK participated in the design of the study and performed the statistical analysis. DT-K produced data for the study from her local centre. AU-K produced data for the study from her local centre. OO produced data for the study from his local centre. RG produced data for the study from her local centre. SB produced data for the study from his local centre. GM produced data for the study from his local centre. SN-A produced data for the study from her local centre. NE-T produced data for the study from her local centre. TD produced data for the study from his local centre. NE produced data for the study from his local centre. CA-H produced data for the study from her local centre. EY produced data for the study from her local centre. BM produced data for the study from her local centre. BK produced data for the study from her local centre. NC produced data for the study from her local centre. OK produced data for the study from his local centre. DI produced data for the study from her local centre. MC produced data for the study from her local centre. SS produced data for the study from her local centre. BY-D produced data for the study from her local centre. SY produced data for the study from her local centre. CA produced data for the study from her local centre. YB produced data for the study from his local centre. YA produced data for the study from her local centre. ST produced data for the study from his local centre. HY produced data for the study from his local centre. HB produced data for the study from his local centre. HAE produced data for the study from his local centre. ND produced data for the study from her local centre. MD produced data for the study from his local centre. SO produced data for the study from his local centre. NS produced data for the study from her local centre. TS produced data for the study from her local centre. ORS produced data for the study from his local centre. SU produced data for the study from his local centre. EsY produced data for the study from her local centre. SK produced data for the study from his local centre. AU participated the design and the coordination of the study. HK participated the design and the coordination of the study. BB participated the design and the coordination of the study. HV participated the design and the coordination of the study. YT participated the design and the coordination of the study. GU participated the design and the coordination of the study. DA participated the design and the coordination of the study. HD participated the design and the coordination of the study. SU participated the design and the coordination of the study. HL participated the design and the coordination of the study. All authors read and approved the final manuscript.
